# Advances in Hydrogen, Carbon Dioxide, and Hydrocarbon Gas Sensor Technology Using GaN and ZnO-Based Devices

**DOI:** 10.3390/s90604669

**Published:** 2009-06-15

**Authors:** Travis Anderson, Fan Ren, Stephen Pearton, Byoung Sam Kang, Hung-Ta Wang, Chih-Yang Chang, Jenshan Lin

**Affiliations:** 1 Department of Chemical Engineering, University of Florida / Gainesville, FL 32611, USA; 2 Department of Materials Science and Engineering, University of Florida / Gainesville, FL 32611, USA; E-Mail: ren@che.ufl.edu (F.R.); 3 Department of Electrical and Computer Engineering, University of Florida / Gainesville, FL 32611, USA; E-Mail: spear@mse.ufl.edu (S.P.)

**Keywords:** GaN, ZnO, gas sensors

## Abstract

In this paper, we review our recent results in developing gas sensors for hydrogen using various device structures, including ZnO nanowires and GaN High Electron Mobility Transistors (HEMTs). ZnO nanowires are particularly interesting because they have a large surface area to volume ratio, which will improve sensitivity, and because they operate at low current levels, will have low power requirements in a sensor module. GaN-based devices offer the advantage of the HEMT structure, high temperature operation, and simple integration with existing fabrication technology and sensing systems. Improvements in sensitivity, recoverability, and reliability are presented. Also reported are demonstrations of detection of other gases, including CO_2_ and C_2_H_4_ using functionalized GaN HEMTs. This is critical for the development of lab-on-a-chip type systems and can provide a significant advance towards a market-ready sensor application.

## 1. Introduction

The Gallium Nitride (GaN) materials system is attracting much interest for commercial applications. Due to the wide-bandgap nature of the material, it is very thermally stable, and electronic devices can be operated to temperatures up to 500 °C. The material is also chemically stable, with the only known wet etchant being molten NaOH or KOH, making it very suitable for operation in chemically harsh environments or in radiation fluxes. Due to the high electron mobility, Nitride-based HEMTs can operate from very high frequency (VHF) through X-band frequencies with higher breakdown voltage, better thermal conductivity, and wider transmission bandwidths than Si or GaAs devices. GaN-based HEMTs can also operate at significantly higher power densities and higher impedance than currently used GaAs devices [1-16].

An overlooked potential application of the AlGaN/GaN HEMT structure is sensors. The high electron sheet carrier concentration of nitride HEMTs is induced by piezoelectric polarization of the strained AlGaN layer and the spontaneous polarization is very large in wurtzite III-nitrides. This provides an increased sensitivity relative to simple Schottky diodes fabricated on GaN layers [17-35]. The gate region can be functionalized so that current changes can be detected for a variety of gases, liquids, and biomolecules. Hydrogen sensors are particularly interesting for the emerging fuel cell vehicle market. There are also applications for detection of combustion gases for fuel leak detection in spacecraft, automobiles and aircraft, fire detectors, exhaust diagnosis and emissions from industrial processes [36-45]. A variety of gas, chemical and health-related sensors based on HEMT technology have been demonstrated with proper surface functionalization on the gate area of the HEMTs, including the detection of hydrogen, mercury ion, prostate specific antigen (PSA), DNA, and glucose [46-49].

Schottky diodes with Pt or Pd gates have been shown to be particularly effective hydrogen sensors. The sensing mechanism is ascribed to the dissociation of the molecular hydrogen on a catalytic metal gate contact, followed by diffusion of the atomic species to the semiconductor interface where it changes the piezoelectric charge in the channel and thus the effective barrier height on Schottky diode structures. This effect has been used in Si, SiC, ZnO and GaN–based Schottky diode combustion gas sensors [50-59].

One of the main demands for such sensors is the ability to selectively detect hydrogen at room temperature in the presence of air. In addition, for most of these applications, the sensors should have very low power requirements and minimal weight. Nanostructures are natural candidates for this type of sensing. One important aspect is to increase their sensitivity for detecting gases such as hydrogen at low concentrations or temperatures, since typically an on-chip heater is used to increase the dissociation efficiency of molecular hydrogen to the atomic form and this adds complexity and power requirements. Previous work has shown that Pd-coated or doped carbon nanotubes (CNTs) become more sensitive to detection of hydrogen through catalytic dissociation of H_2_ to atomic hydrogen [60-62]. ZnO is also an attractive material for sensing applications and nanowires and nanorods in this system have been reported for pH, gas, humidity and chemical sensing [63-66]. These nanostructures could also have novel applications in biomedical science because ZnO is bio-safe [67]. ZnO nanorods are relatively straightforward to synthesize by a number of different methods [67-80].

A couple of problems that arise when considering marketable applications are false alarms and stability. These can be caused by voltage swings in the device or simply by temperature changes altering the current level. A differential pair configuration of AlGaN/GaN HEMT diodes with a built-in control diode has been shown to reduce false alarms [81]. An additional key need for long-term monitoring applications is the availability of stable Ohmic contacts. We have found that boride-based Ohmic contacts on HEMTs show lower contact resistance than Ti/Al/Pt/Au after extended aging at 350 °C [82]. Our test system consists of an environmental chamber with an electrical feed-through for monitoring the device I-V characteristics. Mass flow controllers are used to introduce test gases and nitrogen to the chamber and vary the concentration, and the chamber passes through a furnace for testing at elevated temperature.

## 2. Sensors for Hydrogen

### 2.1. ZnO Nanorods

**Figure 1.**
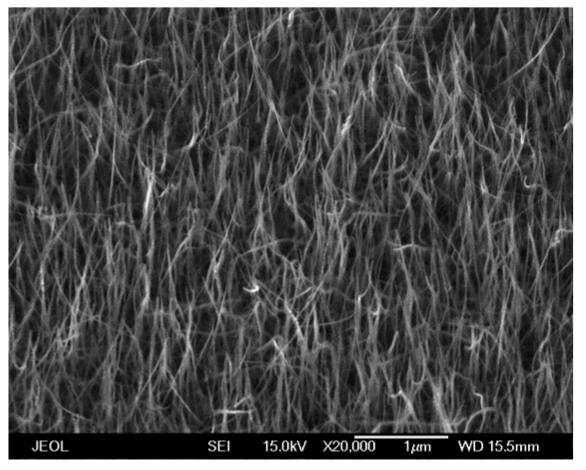
SEM image of ZnO multiple nanorods.

We have developed a consistent process for growing ZnO nanorods. We start by evaporating a thin Au layer (20 Å) and annealing to form islands, which serve as the nucleation sites for the nanorods. The nanorods are then deposited by Molecular Beam Epitaxy (MBE) using high purity Zn metal and an O_3_/O_2_ plasma discharge as the source chemicals. After ∼2 h growth time at 600 °C, we have grown single-crystal nanorods with a typical length around 2∼10 μm and diameter in the range of 30-150 nm. Figure 1 shows a scanning electron micrograph of the as-grown rods. These alone are sensitive to hydrogen, but in some experiments we attempted to increase the sensitivity by sputtering Pd, Pt, Au, Ni, Ag or Ti thin films (∼100 Å thick) to form catalytic metal clusters.

**Figure 2.**
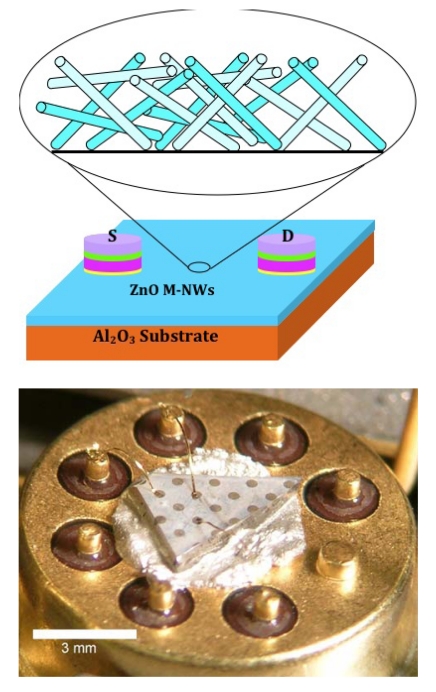
Schematic of contact geometry for multiple nanorod gas sensor (top) and packaged, wire-bonded device for testing (bottom).

Contacts to the multiple nanorods were formed using a shadow mask and sputtering of Al/Ti/Au electrodes. The separation of the electrodes was ∼30 μm. Au wires were bonded to the contact pad for current–voltage (I-V) measurements performed at 25 °C in a range of different atmospheres (N_2_, O_2_ or 10-500 ppm H_2_ in N_2_). A schematic of the resulting sensor is shown in Figure 2 (top) with an optical image of a wire-bonded device shown below. Note that no currents were measured through the discontinuous Au islands and no thin film of ZnO on the sapphire substrate was observed with the growth condition for the nanorods. Therefore the measured currents are due to transport through the nanorods themselves. The I-V characteristics from the multiple nanorods were linear with typical currents of 0.8 mA at an applied bias of 0.5 V.

**Figure 3.**
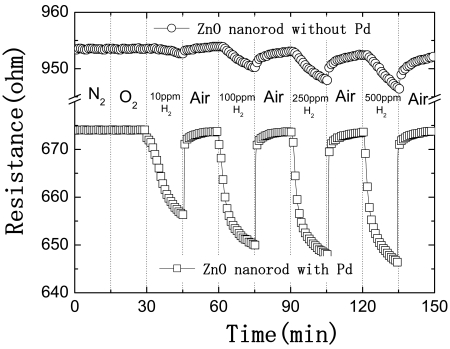
Time dependence of resistance of either Pd-coated or uncoated multiple ZnO nanorods as the gas ambient is switched from N_2_ to various concentrations of H_2_ in air (10-500 ppm).

Figure 3 shows the time dependence of resistance of either Pt-coated or uncoated multiple ZnO nanorods as the gas ambient is switched from N_2_ to various concentrations of H_2_ in air (10-500 ppm) as time proceeds. There is clearly a strong increase (approximately a factor of 5) in the response of the Pd-coated nanorods to hydrogen relative to the uncoated devices. The addition of the Pd appears to be effective in catalytic dissociation of the H_2_ to atomic hydrogen. In addition, there was no response to the presence of O_2_ in the ambient at room temperature, and the relative response of Pt-coated nanorods is a function of H_2_ concentration in N_2_. The Pd-coated CNTs detected hydrogen down to < 10 ppm, with relative responses of > 2.6% at 10 ppm and > 4.2% at 500 ppm H_2_ in N_2_ after 10 min exposure. By comparison, the uncoated devices showed relative resistance changes of ∼0.25% for 500 ppm H_2_ in N_2_ after 10 min exposure and the results were not consistent for lower concentrations. The gas sensing mechanism suggested include the desorption of adsorbed surface hydrogen and grain boundaries in poly-ZnO [83], exchange of charges between adsorbed gas species and the ZnO surface leading to changes in depletion depth [84] and changes in surface or grain boundary conduction by gas adsorption/desorption [85]. It should be noted that hydrogen introduces a shallow donor state in ZnO and this change in near-surface conductivity may also play a role.

**Figure 4.**
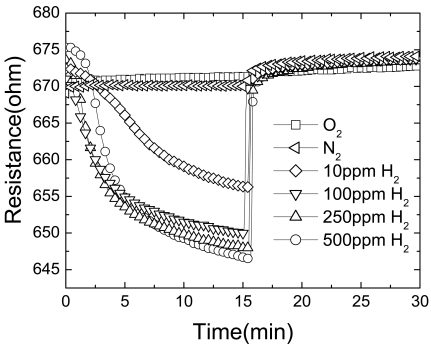
Relative response of Pd-coated nanorods as a function of H_2_ concentration in N_2_.

Figure 4 shows the time dependence of resistance change of Pt-coated multiple ZnO nanorods as the gas ambient is switched from vacuum to N_2_, oxygen or various concentrations of H_2_ in air (10-500 ppm) and then back to air. This data confirms the absence of sensitivity to O_2_. The resistance change during the exposure to hydrogen was slower in the beginning and the rate resistance change reached a maximum at 1.5 min of the exposure time. This could be due to the some of the Pd becoming covered with native oxide and then removed by exposure to hydrogen. Since the available surface Pd for catalytic chemical absorption of hydrogen increased after the removal of oxide, the rate of resistance change increased. However, the Pd surface gradually saturated with the hydrogen and resistance change rate decreased. When the gas ambient switched from hydrogen to air, the oxygen reacted with hydrogen right away, and the resistance of the nanorods changed back to the original value instantly.

**Figure 5.**
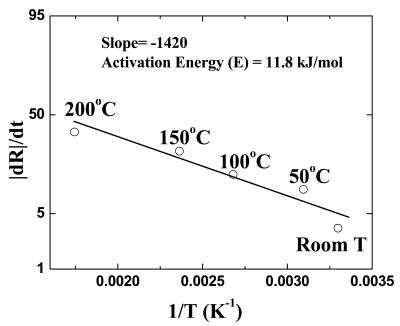
Time dependence of resistance change of Pd-coated multiple ZnO nanorods as the gas ambient is switched from N_2_ to oxygen or various concentrations of H_2_ in air (10-500 ppm) and then back to N_2_.

Figure 5 shows the Arrhenius plot of the rate of nanorod resistance change. The rate of resistance change for the nanorods exposed to the 500 ppm H_2_ in N_2_ was measured at the different temperatures. An activation energy of 12 kJ/mole was extracted from the slope of the Arrehnius plot. This value is larger than that of the typical diffusion process. Therefore the dominant mechanism for this sensing process should be the chemisorption of hydrogen to the Pd surface.

**Figure 6.**
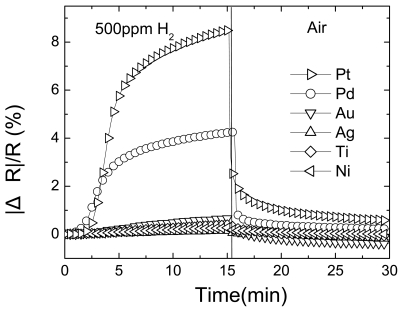
Time dependence of relative resistance response of metal-coated multiple ZnO nanorods as the gas ambient is switched from N_2_ to 500 ppm of H_2_ in air as time proceeds. There was no response to O_2_.

Having established improved sensitivity by coating the nanorods with catalytic metal, we then investigated the effects of different metals to further improve sensitivity and response. Using the same coating procedure described above, we investigated metal coatings of Ti, Ni, Ag, Au, Pt, and Pd. Figure 6 shows the time dependence of relative resistance change of either metal-coated or uncoated multiple ZnO nanorods as the gas ambient is switched from air to 500 ppm of H_2_ in N_2_. These were measured at a bias voltage of 0.5 V. There is a strong enhancement in response with Pd, and to a lesser extent Pt coatings, but the other metals produce little or no change. This is consistent with the known catalytic properties of these metals for hydrogen dissociation. Pd has a higher permeability than Pt but the solubility of H_2_ is larger in the former [86]. Moreover, studies of the bonding of H to Ni, Pd and Pt surfaces have shown that the adsorption energy is lowest on Pt [87].

**Figure 7.**
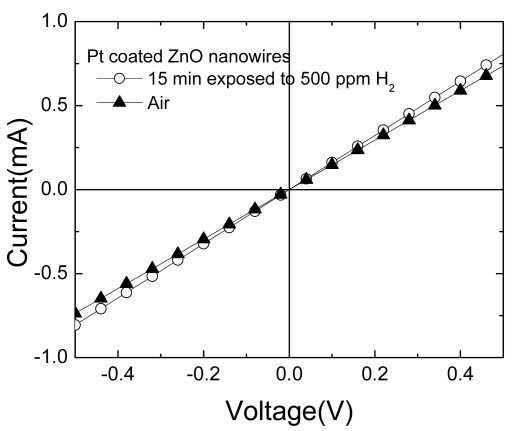
I-V characteristic of Pt-coated nanowires in air and after 15 mins in 500 ppm H_2_ in air.

The power requirements for the sensors were very low, which is a key requirement for a competitive marketable sensor. Figure 7 shows the I-V characteristics measured at 25 °C in both a pure N_2_ ambient and after 15 min in a 500 ppm H_2_ in N_2_ ambient. Under these conditions, the resistance response is 8% and is achieved for a power requirement of only 0.4 mW. This compares well with competing technologies for hydrogen detection such as Pd-loaded carbon nanotubes [60,61].

### 2.2. AlGaN/GaN HEMT

**Figure 8.**
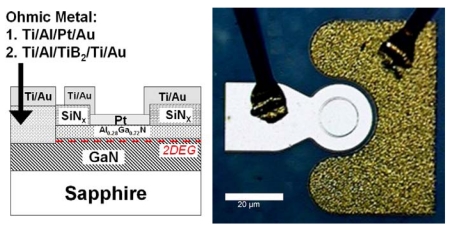
Cross-sectional schematic of completed MOS diode on AlGaN/GaN HEMT layer structure (left) and plan-view photograph of device(right).

The device layer structures were grown on C-plane Al_2_O_3_ substrates by Metal Organic Chemical Vapor Deposition (MOCVD). The layer structure included an initial 2 μm thick undoped GaN buffer followed by a 35 nm thick unintentionally doped Al_0.28_Ga_0.72_N layer. Mesa isolation was achieved by using an inductively coupled plasma system with Ar/Cl_2_ based discharges. The Ohmic contacts were formed by lift-off of sputtered Ti/Al/TiB_2_/Ti/Au, followed by annealing at 850 °C for 45 sec under a flowing N_2_ ambient. A thin (100 Å) Pt Schottky contact was deposited by e-beam evaporation for the Schottky metal. The final step was deposition of e-beam evaporated Ti/Au interconnection contacts. The individual devices were diced and wire-bonded to carriers. These were then placed in our test chamber. Figure 8 shows a schematic of the completed devices and an optical image of a wire bonded device. Mass flow controllers were used to control the gas flow through the chamber, and the devices were exposed to either 100% pure N_2_, or H_2_ concentrations of 500 ppm down to 1 ppm in N_2_ and temperatures from 25 to 500 °C.

**Figure 9.**
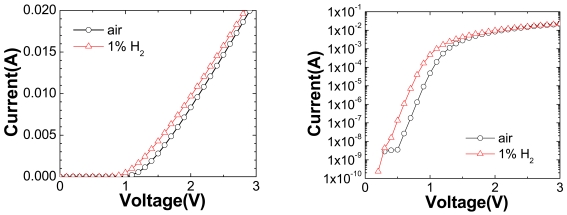
I-V characteristics in linear (top) or log (bottom) form of Pt-gated diode measured in air or 1% hydrogen ambient at 25 °C.

Figure 9 shows the linear (top) and log scale (bottom) forward current-voltage (I-V) characteristics at 25 °C of the HEMT diode, both in air and in a 1% H_2_ in air atmosphere. For these diodes, the current increases upon introduction of the H_2_, through a lowering of the effective barrier height. The H_2_ catalytically decomposes on the Pt metallization and diffuses rapidly to the interface where it forms a dipole layer. The differential change in forward current upon introduction of the hydrogen into the ambient is ∼1 mA over the voltage range examined.

**Figure 10.**
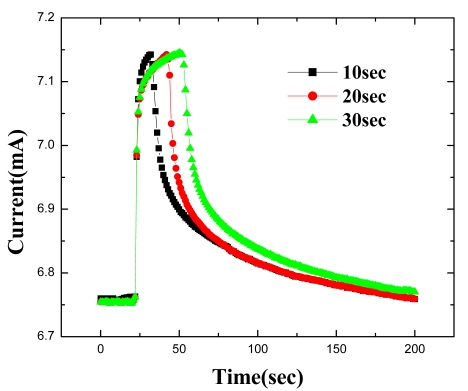
Time response at 25 °C of MOS-HEMT based diode forward current at a fixed bias of 2V when switching the ambient from N_2_ to 10%H_2_ /90%N_2_ for periods of 10, 20 or 30 sec and then back to pure N_2._

To test the time response of the sensors, a 10% H_2_/90% N_2_ ambient was switched into the chamber through a mass flow controller for periods of 10, 20 or 30 seconds and then switched back to pure N_2._ Figure 10 shows the time dependence of forward current at a fixed bias of 2 V under these conditions. The response of the sensor is rapid (< 1 sec), with saturation taking almost the full 30 seconds. Upon switching out of the hydrogen–containing ambient, the forward current decays exponentially back to its initial value. This time constant is determined by the volume of the test chamber and the flow rate of the input gases and is not limited by the response of the diode itself.

**Figure 11.**
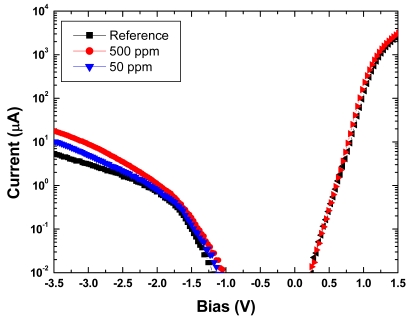
Forward and reverse bias plot of diode current in varying atmospheres.

To further study response and detection limits, devices were tested under both forward and reverse bias conditions at room temperature (25 °C) in a nitrogen atmosphere at hydrogen concentrations ranging from 500 to 5 ppm, controlled by diluting the gas mix with nitrogen using mass flow controllers. There was again an increase in current under both forward and reverse bias conditions upon exposure to hydrogen, as shown in Figure 11. This is consistent with previously discussed mechanisms in which the hydrogen molecules dissociate into hydrogen atoms through the catalytic action of the Pt gate contact, and diffuse to the Pt/AlGaN interface [88,89]. The hydrogen atoms form a dipole layer, lower the Schottky barrier height, and increase net positive charges on the AlGaN surface as well as negative charges in the 2DEG channel. The calculated barrier height decrease for 500 ppm and 100 ppm hydrogen is 5 meV and 1 meV, respectively. The ideality factors were calculated to be 1.25 and 1.23 in 500 and 100 ppm hydrogen, respectively, compared to 1.26 in 100% nitrogen.

**Figure 12.**
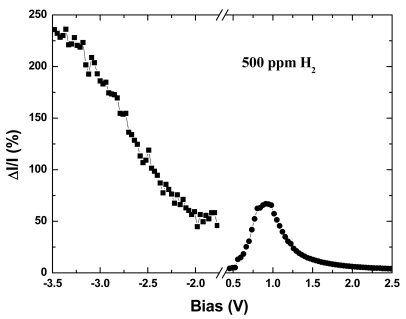
Percentage change in current as a function of bias at 500 ppm H_2_.

However, a plot of hydrogen sensitivity (defined as the drain current change over the initial drain current) versus bias voltage shows different characteristics for forward and reverse bias polarity conditions at 500 ppm of H_2_, as shown in Figure 12. For the forward bias condition, there is a maximum sensitivity obtained around 1 V and further increase of bias voltage reduces the sensitivity. The sensitivity for the reverse bias condition is quite different and it increases proportionally to the bias voltage. We have proposed the following mechanism for the change in sensitivity under forward and reverse bias conditions: (1) The initial increase in the sensitivity is due to the Schottky barrier height reduction. (2) Further increase in forward bias allows electrons to flow across the Schottky barrier. These excess electrons bind with H^+^, form atomic hydrogen, and gradually destroy the dipole layer at the interface, therefore losing the hydrogen detection sensitivity. (3) For the reverse bias condition, electrons given away by the hydrogen atom may be swept across the depletion region. At higher reverse bias voltage, a higher driving force is applied to the electrons to move across the depletion region. Thus the dipole layer is amplified at the Pt/AlGaN interface for higher reverse bias voltage. Due to this dipole layer amplification, the detection sensitivity is enhanced at higher reverse bias voltage.

**Figure 13.**
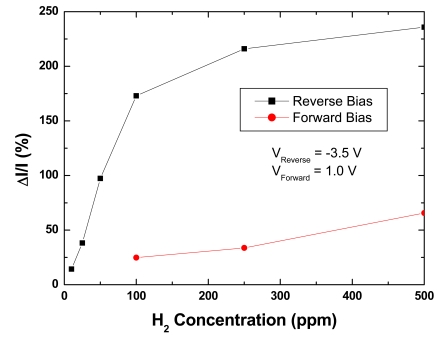
Percentage change in current as a function of hydrogen concentration under both forward and reverse bias.

The detection sensitivity as a function of hydrogen concentration is shown in Figure 13. It is clear that the diodes are much more sensitive under reverse bias conditions. A detection limit of 100 ppm is achieved under forward bias, but the reverse bias detection limit is an order of magnitude lower, 10 ppm. The change in current at 10 ppm is 14% and over 200% at 500 ppm under reverse bias conditions, where forward bias operation results in changes of 25-75% over the 100-500 ppm range. This is consistent with published reports indicating improved sensitivity under reverse bias [90]. The reliability of the hydrogen sensor may be quite different under the two bias voltage polarities, since different degradation mechanisms in GaN devices are accelerated by either the presence of high voltage depletion regions (reverse bias) or current injection (forward bias) [91].

## 3. Sensors for CO_2_

A sensor for CO_2_ gas has many potential applications, for example monitoring global warming, indoor air quality control, process control in fermentation, and the medical field [92-95]. A particular application that we have focused on is quantification of CO_2_ concentrations in patient's exhaled breath. This is a critically important measurement that allows health care providers to assess adequacy of ventilation and circulation in subjects. The most common approach for CO_2_ detection is based on non-dispersive infrared (NDIR) sensors, which are the simplest of the spectroscopic sensors [96-99]. The best detection limits for the NDIR sensors in the range 20-10,000 ppm. The key components of NDIR sensors are an infrared source, a light tube, an interference filter, and an infrared detector, thus the technology is quite limited by physical size and power consumption. In recent years, monomers or polymers containing an amino-group, such as tetrakis (hydroxyethyl)ethylenediamine, tetraethylene-pentamine and polyethyleneimine (PEI) have been used as coatings of surface acoustic wave transducers to overcome these limitations [100-104]. PEI has also been used as a coating on carbon nanotubes for CO_2_ sensing by measuring the conductivity of nanotubes upon exposure to CO_2_ [105-108].

In this section, we report the design and fabrication of a chemically functionalized AlGaN/GaN HEMT device for CO_2_ sensing. Specific sensitivity can be achieved by employing a CO_2_ recognition layer of PEI/starch on the gate area of the HEMT. The effect of temperature and CO_2_ concentration on the sensing sensitivity was investigated.

**Figure 14.**
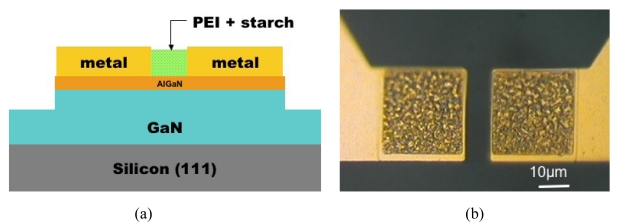
(a) Schematic of AlGaN/GaN HEMT based CO_2_ sensor (b) plan view photomicrograph of PEI/starch functionalized HEMT CO_2_ sensor.

The device fabrication followed the standard procedure outlined previously. In this case the HEMT structure consisted of an undoped GaN buffer, a thin undoped Al_0.3_Ga_0.7_N spacer, and a Si-doped Al_0.3_Ga_0.7_N cap layer. The epi-layers were grown by Metal-Organic Chemical Vapor Deposition (MOCVD) on silicon (111) substrates. Mesa isolation was again achieved by ICP etching, ohmic contacts were formed using annealed Ti/Al/Pt/Au, interconnects formed using Ti/Au metallization, and the gate was left open for this experiment. Polymethyl methacrylate (PMMA) was used to encapsulate the sample, with only the gate region opened for selective functionalization using e-beam lithography. A mixture of PEI and starch was used for the CO_2_-sensitive layer and the mixture was applied via spin-coating. The completed device was then diced, wire-bonded, and mounted on the previously described test apparatus. A plan view photomicrograph of a completed device and a schematic cross-section of the device are shown in Figure 14. The gas exposure sequence in this case consisted of repeated exposures to gas with different CO_2_ concentration balanced with pure N_2_.

**Figure 15.**
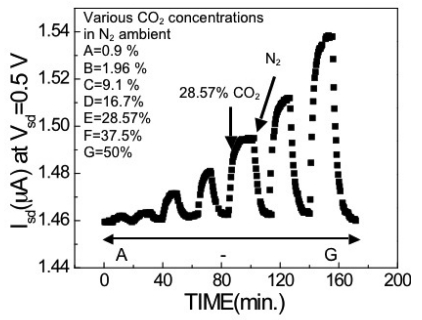
Drain current of PEI/starch functionalized HEMT sensor measured at fixed source-drain during the exposure to different CO_2_ concentration ambients. The drain bias voltage was 0.5 V and measurements were conducted at 108 °C.

The interaction between CO_2_ and amino group-containing compounds with the influence of water molecules is based on an acid-base reaction. The purpose of adding starch into the PEI in our experiment was to enhance the absorption of the water molecules into the PEI/starch thin film. Several possible reaction mechanisms have been suggested. The key reaction was that primary amine groups, –NH_2_, on the PEI main chain reacted with CO_2_ and water forming −NH_3_^+^ ions and the CO_2_ molecule became OCOOH ions, changing the polarity of the PEI main chain. This will then affect the surface charge on the gate region of the HEMT, thus changing the carrier density in the 2DEG, effectively modulating the source-drain current in the same way that a real gate would. Figure 15 shows the drain current of PEI/starch functionalized HEMT sensors measured exposed to different CO_2_ concentration ambients.

The measurements were conducted at 108 °C and a fixed source-drain bias voltage of 0.5 V. The reason for choosing this temperature will be discussed later. The current increased with the introduction of CO_2_ gas due to the increase in net positive charge on the gate area, which induced electrons in the 2DEG channel. The response to CO_2_ gas has a wide dynamic range from 0.9% to 50%. Higher CO_2_ concentrations were not tested because there is little interest in these for medical related applications. The response times were on the order of 100 seconds. The signal decay time was slower than the rise time and was due to the longer time required to purge CO_2_ out from the test chamber.

**Figure 16.**
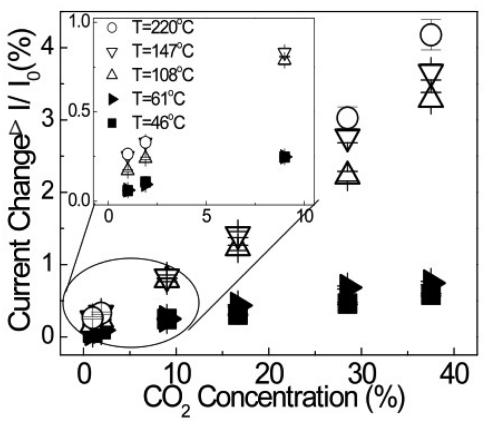
The drain current changes of HEMT sensor as a function of CO_2_ concentration. The inset is the current change of the sensors as function of lower CO_2_ concentrations (0.9-10%).

**Figure 17.**
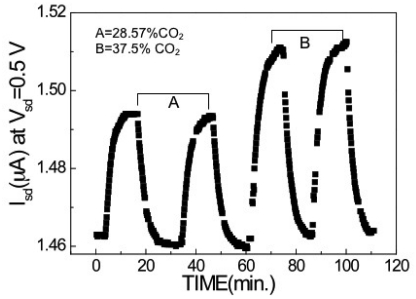
Drain current of PEI/starch functionalized HEMT sensor as a function of time measured at a drain bias voltage of 0.5 V and at 108 °C.

The ambient temperature is critical to the sensitivity of this device. Figure 16 shows the percentage of drain current change as a function of CO_2_ concentration from 0.9 to 40% at five different testing temperatures ranging from 46 to 220 °C. The insert of the figure shows an enlargement of the drain current changes at lower concentrations. The drain current changes increased monotonically to the CO_2_ concentration for all the tested temperatures. However, the HEMT sensors showed greater sensitivity at the higher testing temperatures. There is a particularly noticeable increase in sensitivity from the sensors tested at 61 °C to those tested at 108 °C. This difference is likely due to an increase in the reaction rate between amine groups and CO_2_ as well as the diffusion of CO_2_ molecules into the PEI thin film. Another important figure of merit for sensors is the reversibility and reproducibility, shown in Figure 17. The sensor was exposed to two different CO_2_ concentrations twice at 28.5 and 37.5%, respectively. Similar responses were obtained for the same CO_2_ concentration for both cases, and the current recovered back to the baseline level easily in N_2_.

## 4. Sensors for Ethylene

Ethylene is a particularly interesting case for gas sensors because the strong double bonds prove difficult to dissociate on a catalytic surface at modest temperatures. This is where the high temperature capabilities of wide bandgap materials become very relevant. In this section we will report on detection of C_2_H_4_ using bulk ZnO schottky diodes and AlGaN/GaN MOS-diodes, with reasonable sensitivity demonstrated above 150 °C.

### 4.1. Bulk ZnO

**Figure 18.**
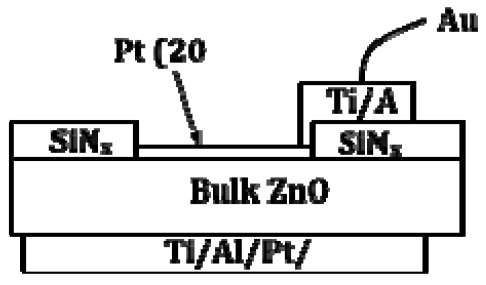
Schematic of AlGaN/GaN MOS diode (top) and bulk ZnO Schottky diode structure (bottom).

The bulk ZnO crystals from Cermet, Inc. showed electron concentration of 9 × 10^16^ cm^-3^ and an electron mobility of 200 cm^2^/V. s. at room temperature from van der Pauw measurements. Ohmic contacts were formed on the back (O-face) of the substrates by depositing full area Ti/Al/Pt/Au by e-beam evaporation followed by annealing at 200 °C for 1 min in N_2_ ambient. The front face was coated with plasma-enhanced chemical vapor deposited SiN_X_ at 100°C and windows opened by wet etching so that a thin (20 nm) layer of Pt could be deposited by e-beam evaporation to serve as the sensing metal. The final Ti/Au interconnect contacts were deposited, followed by dicing, wire-bonding, and mounting to the test apparatus. Figure 18 shows a schematic of the completed device.

**Figure 19.**
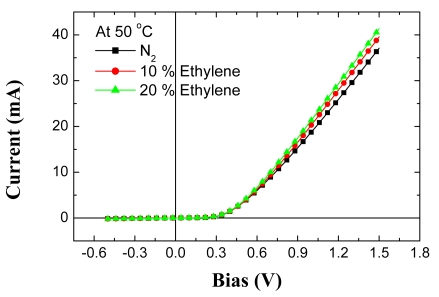

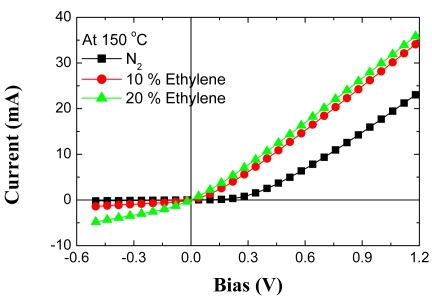
I-V characteristics at 50 °C (top) or 150 °C (bottom) of Pt/ZnO diodes measured in different ambients.

**Figure 20.**
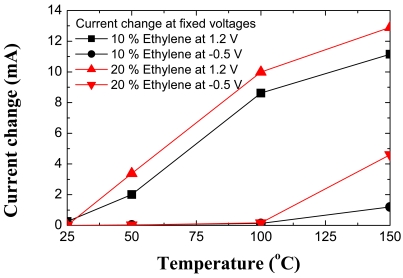
Change in current at a fixed bias as a function of measurement temperature in different percentages of C_2_H_4_/N_2_ ambients.

Figure 19 shows the I-V characteristics at 50 and 150 °C of the Pt/ZnO diode both in pure N_2_ and in ambients containing various concentrations of C_2_H_4_. At a given forward or reverse bias, the current increases upon introduction of the C_2_H_4_, through a lowering of the effective barrier height. The sensing mechanism is once again the decomposition of the C_2_H_4_ on the catalytic Pt surface, followed by diffusion to the underlying interface with the ZnO. As in hydrogen sensors, the atomic hydrogen forms an interfacial dipole layer that will reduce the Schottky barrier height, which will be manifested by a change in the DC I-V characteristics of the diode. The recovery was many orders of magnitude longer than for Pt/GaN diodes measured under the same conditions in the same chamber, suggesting that hydrogen diffuses into the ZnO lattice much more than it does for GaN. The changes in current at fixed bias or bias at fixed current were larger for the ZnO diodes than for AlGaN/GaN MOS diodes, which will be discussed in the next section, because of this additional detection mechanism, as shown in Figure 20. Note that the changes in these parameters are approximately an order of magnitude larger at 150 °C, suggesting that testing at even higher temperature will further improve sensitivity, but the ZnO diodes were not thermally stable above ∼300 °C.

### 4.2. AlGaN/GaN MOS-Diode

**Figure 21.**
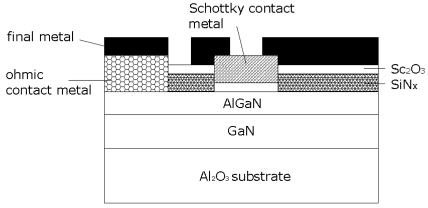
Schematic of AlGaN/GaN MOS diode.

AlGaN/GaN layer structures were grown on C-plane Al_2_O_3_ substrates by Metal Organic Chemical Vapor Deposition (MOCVD). The layer structure included an initial 2 μm thick undoped GaN buffer followed by a 35 nm thick unintentionally doped Al_0.28_Ga_0.72_N layer. The sheet carrier concentration was ∼1×10^13^ cm^-2^ with a mobility of 980 cm^2^/V-s at room temperature. Device isolation was achieved with 2,000 Å plasma enhanced chemical vapor deposited SiNx. The ohmic contacts was formed by annealing e-beam deposited Ti/Al/Pt/Au. 400 Å Sc_2_O_3_ was deposited as a gate dielectric through a contact window of SiNx layer. Before oxide deposition, the wafer was exposed to ozone for 25 minutes. It was then heat in-situ at 300 °C cleaning for 10mins inside the growth chamber. 100 Å Sc_2_O_3_ was deposited on AlGaN/GaN by rf plasma-activated MBE at 100 °C using elemental Sc and plasma O_2_ [109,110]. The thin Pt Schottky contact was deposited on the top of Sc_2_O_3_. Then, Ti/Au interconnection contacts were deposited, followed by dicing and wire-bonding. The devices were then mounted in the test chamber previously described. Figure 21 shows a schematic of the completed device.

**Figure 22:**
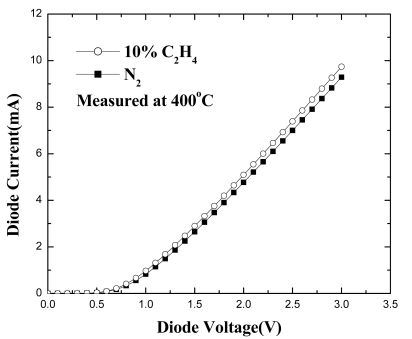
Forward I-V characteristics of MOS-HEMT based diode sensor at 400 °C measured under pure N_2_ or 10% C_2_H_4_ /90% N_2_ ambients.

Figure 22 shows the forward diode current-voltage (I-V) characteristics at 400 °C of the Pt/Sc_2_O_3_/AlGaN/GaN MOS-HEMT diode both in pure N_2_ and in a 10% C_2_H_4_/90%N_2_ atmosphere. The reason for testing at 400 °C will be discussed later. At a given forward bias, the current increases upon introduction of the C_2_H_4_. The sensing mechanism follows the same path as the hydrogen sensor - Hydrogen either decomposed from C_2_H_4_ in the gas phase or chemisorbed on the Pt Schottky contacts then decomposed and released hydrogen. The hydrogen diffused rapidly though the Pt metallization and the underlying oxide to the interface where it forms a dipole layer and lowered the effective barrier height.

**Figure 23:**
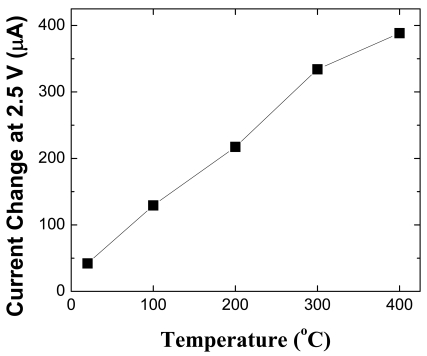
Change in MOS diode forward current at fixed forward bias of 2.5V as a function of temperature for measurement in 10% C_2_H_4_/90% N_2_.

Figure 23 shows both the change in current at fixed bias as a function of temperature for the MOS diodes when switching from a 100% N_2_ ambient to 10% C_2_H_4_/90% N_2._ As the detection temperature is increased, the response of the MOS-HEMT diodes increases due to more efficient cracking of the hydrogen on the metal contact. Note that the change in current is quite large and readily detected.

## 5. Conclusions and Future Work

ZnO nanorods appear well-suited to detection of ppm concentrations of hydrogen at room temperature. In particular, Pt-coated nanowires demonstrate a superior response compared to other metals. The recovery characteristics are fast upon removal of hydrogen from the ambient. The primary advantages of ZnO nanorods are that they can be placed on cheap transparent substrates such as glass, making them attractive for low-cost sensing applications and can operate at very low power conditions.

AlGaN/GaN HEMT diodes appear well-suited to combustion gas sensing applications. The changes in forward current are approximately double those of simple GaN Schottky diode gas sensors tested under similar conditions and suggest that integrated chips involving gas sensors and HEMT-based circuitry for off-chip communication are feasible in the AlGaN/GaN system. Devices show improved sensing capabilities under reverse bias polarity. Due to the amplification of the dipole layer at the interface under reverse bias conditions instead of screening under forward bias conditions, the sensitivity of hydrogen detection is higher under the reverse bias conditions. By using reverse bias condition combined with the improved stability from boride contacts, the overall stability of the GaN system is very attractive for long-term applications requiring high sensitivity. Devices show greatly improved current stability under field conditions with use of Ti/Al/TiB_2_/Ti/Au contacts replacing the more conventional Ti/Al/Pt/Au. Combined with the superior thermal stability of these boride-based contacts, this metallization system appears attractive for sensors for long-term monitoring applications.

We have also demonstrated PEI/starch functionalized HEMT sensors for CO_2_ detection with a wide dynamic range from 0.9% to 50%. The sensors were operated at low bias voltage (0.5 V) for low power consumption applications. The sensors exhibited greater sensitivity at the testing temperature higher than ∼100 °C. The sensors showed good repeatability. This electronic detection of CO_2_ gas is a significant step towards a compact sensor chip, which can be integrated with a commercially available hand-held wireless transmitter to realize a portable, fast and highly sensitive CO_2_ sensor.

AlGaN/GaN MOS-HEMT diodes and bulk ZnO Schottky diodes appear well-suited to detection of C_2_H_4_. The former have a larger temperature range of sensitivity, but the absolute changes in voltage or current are larger with the ZnO diodes. The introduction of hydrogen shallow donors into the near-surface region of the ZnO is a plausible mechanism for the non-recovery of the I-V characteristics at room temperature.
